# Co-ingestion of whey protein hydrolysate with milk minerals rich in calcium potently stimulates glucagon-like peptide-1 secretion: an RCT in healthy adults

**DOI:** 10.1007/s00394-019-02092-4

**Published:** 2019-09-17

**Authors:** Yung-Chih Chen, Harry A. Smith, Aaron Hengist, Oliver J. Chrzanowski-Smith, Ulla Ramer Mikkelsen, Harriet A. Carroll, James A. Betts, Dylan Thompson, John Saunders, Javier T. Gonzalez

**Affiliations:** 1 Department for Health, University of Bath, Bath, BA2 7AY UK; 2 Department of Physical Education, National Taiwan Normal University, Taipei, Taiwan; 3 Arla Foods Ingredients Group P/S, Viby J, Denmark; 4 Rowett Institute, University of Aberdeen, Aberdeen, UK; 5 Royal United Hospitals Bath NHS Foundation Trust, Bath, UK

**Keywords:** Incretins, Calcium, Protein, Metabolism, Peptide tyrosine tyrosine, Gastric inhibitory polypeptide, Postprandial

## Abstract

**Purpose:**

To examine whether calcium type and co-ingestion with protein alter gut hormone availability.

**Methods:**

Healthy adults aged 26 ± 7 years (mean ± SD) completed three randomized, double-blind, crossover studies. In all studies, arterialized blood was sampled postprandially over 120 min to determine GLP-1, GIP and PYY responses, alongside appetite ratings, energy expenditure and blood pressure. In study 1 (*n *= 20), three treatments matched for total calcium content (1058 mg) were compared: calcium citrate (CALCITR); milk minerals rich in calcium (MILK MINERALS); and milk minerals rich in calcium plus co-ingestion of 50 g whey protein hydrolysate (MILK MINERALS + PROTEIN). In study 2 (*n *= 6), 50 g whey protein hydrolysate (PROTEIN) was compared to MILK MINERALS + PROTEIN. In study 3 (*n *= 6), MILK MINERALS was compared to the vehicle of ingestion (water plus sucralose; CONTROL).

**Results:**

MILK MINERALS + PROTEIN increased GLP-1 incremental area under the curve (iAUC) by ~ ninefold (43.7 ± 11.1 pmol L^−1^ 120 min; *p *< 0.001) *versus* both CALCITR and MILK MINERALS, with no difference detected between CALCITR (6.6 ± 3.7 pmol L^−1^ 120 min) and MILK MINERALS (5.3 ± 3.5 pmol L^−1^ 120 min; *p *> 0.999). MILK MINERALS + PROTEIN produced a GLP-1 iAUC ~ 25% greater than PROTEIN (*p *= 0.024; mean difference: 9.1 ± 6.9 pmol L^−1^ 120 min), whereas the difference between MILK MINERALS versus CONTROL was small and non-significant (*p *= 0.098; mean difference: 4.2 ± 5.1 pmol L^−1^ 120 min).

**Conclusions:**

When ingested alone, milk minerals rich in calcium do not increase GLP-1 secretion compared to calcium citrate. Co-ingesting high-dose whey protein hydrolysate with milk minerals rich in calcium increases postprandial GLP-1 concentrations to some of the highest physiological levels ever reported. Registered at ClinicalTrials.gov: NCT03232034, NCT03370484, NCT03370497.

**Electronic supplementary material:**

The online version of this article (10.1007/s00394-019-02092-4) contains supplementary material, which is available to authorized users.

## Introduction

Glucagon-like peptide-1 (GLP-1) is a gut hormone involved in metabolism, insulin secretion, appetite, and angiogenesis [1–3], and it is thought to contribute to the preservation of metabolic health. For example, increasing postprandial GLP-1 concentrations can protect against glucose intolerance during weight gain in rodents [4]. Consequently, strategies such as bariatric surgery, GLP-1 analogues and dipeptidyl peptidase-IV inhibitors (that increase the activity of GLP-1) to augment circulating GLP-1 availability/action are of great interest for the prevention/treatment of obesity and obesity-related diseases [1]. Whilst these surgical and pharmacological strategies hold some potential to elevate GLP-1 availability/action and thus improve metabolic health and or weight loss, many strategies are either not cost-effective for large-scale use and/or carry risks of harmful or undesirable side-effects [1, 5]. Therefore, nutritional strategies to augment GLP-1 availability may provide an attractive additional or alternative option to current approaches.

Nutrition potently regulates enteroendocrine cell action and therefore gut hormone secretion. For example, the ingestion of protein increases plasma GLP-1 availability [6, 7]. The mechanism(s) by which dietary protein stimulates GLP-1 availability is thought to involve direct stimulation by amino acids of the calcium-sensing receptor expressed on L-cells in the intestine [8]. Importantly, it has now been shown across in vitro [9, 10], ex vivo [8] and in vivo rodent models [11] that the calcium-sensing receptor regulates gut hormone release and is responsive to physiologically relevant extracellular calcium and amino acid concentrations across fasting to postprandial concentration range. However, to date, no study has demonstrated the synergistic effect of protein and calcium ingestion on GLP-1 availability in humans.

The role of dietary calcium in gut hormone secretion is being increasingly revealed. Both acute and chronic supplementation with calcium has been shown to augment plasma GLP-1 availability in humans [12, 13], however not all studies have shown an effect of calcium on plasma GLP-1 availability [7, 14, 15]. This discrepancy could be explained (in part) by differences in the type of calcium and/or co-ingestion with other nutrients, leading to variable calcium concentrations in the ileum, where GLP-1 is primarily secreted [16]. Since most calcium is absorbed in the duodenum/jejunum, oral calcium ingestion does not necessarily achieve ileal calcium concentrations in humans that maximize potential for protein-induced GLP-1 secretion [8, 16, 17]. Since there is evidence that milk-based sources of calcium (e.g., calcium phosphate and the associated minerals that are co-ingested with milk) are more slowly absorbed than other sources of calcium, such as calcium citrate [18, 19], it could be hypothesized that milk minerals rich in calcium expose the ileum to greater concentrations of calcium than calcium citrate, leading to increased GLP-1 secretion, but this remains to be tested.

Accordingly, the collective aim of this series of studies was to assess whether milk sources of calcium increase plasma GLP-1 availability to a greater extent than calcium citrate, and whether calcium-stimulated GLP-1 availability is dependent on the co-ingestion of protein. Since dietary calcium and protein have also been implicated in the secretion of other gut hormones (e.g., glucose-dependent insulinotropic polypeptide, GIP and peptide tyrosine–tyrosine, PYY), and in metabolism, appetite and blood pressure, we also aimed to assess the effects of calcium (type) and protein co-ingestion on GIP and PYY availability, energy expenditure and glycaemia, appetite ratings and blood pressure.

## Methods

### Study design

The present project comprised a series of three acute experiments with identical outcome variables and the recruitment of participants used identical inclusion and exclusion criteria. The only differences between experiments were the treatment conditions and number of participants, as described below. Each experiment was registered at ClinicalTrials.gov (IDs: NCT03232034; NCT03370484; NCT03370497) and was conducted in a randomized (randomization performed by J.T.G. using an online program, randomizer.org), double-blind (the lead investigator Y.C.C and participants were blinded during interventions) crossover design at the University of Bath, UK.

#### Study 1

Study 1 comprised three trials: (1) calcium citrate [4380 mg to provide 1000 mg calcium; CALCITR (NOW foods, Bloomingdale, IL, USA)]; (2) calcium-enriched milk mineral supplement [3745 mg to provide 1000 mg calcium; MILK MINERALS; Capolac^®^ (Arla Foods Ingredients, Viby J, Denmark); or (3) calcium-enriched milk mineral supplement (2050 mg to correct for calcium content in protein) plus whey protein hydrolysate [58.8 g providing 50 g protein and 453 mg calcium; MILK MINERALS + PROTEIN; Lacprodan^®^ DI-3065; (Arla Foods Ingredients, Viby J, Denmark)]. Each of these drinks also contained 500 mL of water and artificial sweetener [80 mg sucralose (MyProtein, Northwich, UK); Table 1]. This quantity of sucralose was not expected to stimulate gut hormone secretion, since ingestion of either 80 mg or 800 mg sucralose does not alter incretin hormone or glucose responses [20]. The calcium content of the supplements and of the tap water was independently verified using a commercially available assay (abcam, Cambridge, UK). The day-to-day variation in the calcium content of the tap water was < 15 mg and is, therefore, unlikely to have been sufficient to alter the responses observed. Whilst the MILK MINERALS contained a variety of additional nutrients compared to CALCITR, these were relatively minor (protein, lactose and fat all < 0.5 g and sodium, magnesium, chloride and potassium all < 90 mg). Therefore, the primary nutritional difference is the phosphorus content of MILK MINERALS (Table 1). These products were batch-tested by the manufacturer to confirm the nutritional composition.

**Table 1:** Nutritional composition of each treatment

Ingredient	Treatment				
	CONTROL^a^	CALCITR^b^	MILK MINERALS^c^	PROTEIN^d^	MILK MINERALS + PROTEIN
Energy (kJ)	< 1	< 1	< 5	863	858
Water (mL)	500	500	500	500	500
Sucralose (mg)	80	80	80	80	80
Calcium (mg)	58	1058	1058	453	1058
Phosphorus (mg)	< 1	< 1	551	686	686
Magnesium (mg)	< 1	< 1	26	26	26
Protein (g)	< 0.1	< 0.1	< 0.1	50	50
Carbohydrate (g)	< 0.1	< 0.1	0.3	1.8	1.5
Fat (g)	< 0.1	< 0.1	< 0.1	< 0.1	< 0.1

^a^CONTROL, vehicle of ingestion

^b^CALCITR, calcium citrate

^c^MILK MINERALS, milk minerals rich in calcium

^d^PROTEIN, whey protein hydrolysate

#### Study 2

Study 2 aimed to assess whether the addition of calcium-enriched milk minerals rich in calcium supplement to whey protein hydrolysate enhances gut hormone secretion, compared to the ingestion of whey protein hydrolysate alone. This experiment involved two trials: PROTEIN (58.8 g to provide 50 g protein) and MILK MINERALS + PROTEIN (2050 mg calcium-enriched milk mineral supplement plus 58.8 g whey protein hydrolysate powder to provide 1000 mg calcium plus 50 g protein), whereby the PROTEIN condition included ingestion of 50 g whey protein hydrolysate, mixed in 500 mL water and artificial sweetener (80 mg sucralose; Table 1). Since the whey protein hydrolysate already contained 453 mg calcium, the difference in calcium content between MILK MINERALS + PROTEIN versus PROTEIN, was ~ 600 mg (Table 1).

#### Study 3

The third and final study in this series comprised two trials: CONTROL and MILK MINERALS to establish the effect of calcium-enriched milk minerals rich in calcium supplement ingestion in the absence of protein, on gut hormones responses (Table 1). The calcium content in the CONTROL condition is the background calcium present in the drinking water of the laboratory.

### Participants

Twenty healthy adult men and women (Table 2) were recruited via word-of-mouth and poster advertisement at the University of Bath. All participants completed the treatments comprising study 1. Of this full sample, two subgroups (each *n* = 6) were randomly allocated additional treatments (PROTEIN or CONTROL) for studies 2 and 3 (CONSORT checklist is provided as Supplementary Online Material). Exclusion criteria included: weight instability as defined by > 3% change in body mass in the previous 3 months; any previous or current metabolic, cardio-pulmonary or musculoskeletal diseases; smoking within the last 4 months; not between the ages of 18–65 years; a body mass index (BMI) below 18.5 kg/m^2^ or above 30 kg/m^2^; planned to change lifestyle (diet and/or physical activity) during the study period; or were not willing to refrain from alcohol containing drinks or unaccustomed exercise 1 day before the laboratory sessions. The study protocols were approved by University of Bath, Research Ethics Approval Committee for Health (REACH) (reference number: EP 16/17 164). All participants provided written, informed consent prior to participation in the study, which was conducted in accordance with the Declaration of Helsinki.

**Table 2:** Participant characteristics

	Study 1	Study 2	Study 3
Sample size (of which female)	20 (6)	6 (2)	6 (2)
Age (years)	26 ± 7	25 ± 4	24 ± 4
Body mass (kg)	73.9 ± 9.8	72.5 ± 7.0	71.2 ± 9.3
Body mass index (kg m^−2^)	23.7 ± 2.4	23.8 ± 2.5	22.8 ± 2.0
Waist circumference (cm)	80 ± 9	77 ± 5	74 ± 6
Hip circumference (cm)	99 ± 6	98 ± 3	98 ± 2
Resting metabolic rate (MJ day^−1^)	7.06 ± 0.95	6.86 ± 0.86	6.89 ± 0.85
Fasting RER^a^ ($\dot{V}{\text{CO}}_{ 2} :\dot{V}{\text{O}}_{ 2}$)	0.88 ± 0.04	0.86 ± 0.02	0.88 ± 0.06
Systolic blood pressure (mmHg)	115 ± 6	113 ± 7	115 ± 5
Diastolic blood pressure (mmHg)	73 ± 4	71 ± 3	72 ± 3

Values are mean ± SD. *n* = 20 for study 1. *n* = 6 for studies 2 and 3

*RER* respiratory exchange ratio

### Pre-trial standardization

Participants were asked to record their diet and physical activity for 24 h prior to the first trial and were asked to replicate this diet and physical activity pattern for 24 h prior to all subsequent trials. Tea, coffee and alcohol were not allowed 24 h before the trials and participants were asked to refrain from any vigorous physical activity/exercise 24 h before the trials (confirmed verbally with participants upon subsequent trials). Women who were not on hormonal contraceptives were tested during the follicular phase of the menstrual cycle (3–12 days after the first menses). For women taking hormonal contraceptives, and all men the wash-out period between trials was between 48 h and 7 days.

### Trial days

Participant reported to the laboratory at the University of Bath between 08:00–09:00 h following a 10–12-h overnight fast (standardized within participants). After measurement of body mass, waist and hip circumference, participants rested on a bed for 10 min before determination of blood pressure and resting metabolic rate (RMR) via 5-min samples of expired gas using the Douglas bag technique [21]. To allow for participants to become accustomed to the mouthpiece, this was given to participants 5 min prior to each sample collection. Barometric pressure was 734 ± 5 mmHg, 735 ± 5 mmHg, 734 ± 6 mmHg, 735 ± 6 mmHg and 736 ± 3 mmHg on CALCITR, MILK MINERALS, MILK MINERALS + PROTEIN, PROTEIN and CONTROL trials, respectively. Ambient temperature was 23 ± 1 °C, 22 ± 1 °C, 23 ± 1 °C, 22 ± 1 °C and 23 ± 1 °C on CALCITR, MILK MINERALS, MILK MINERALS + PROTEIN, PROTEIN and CONTROL trials, respectively.

Arterialized venous blood samples were obtained by catheterization of a pre-heated dorsal hand vein as previously described [22]. After a baseline blood sample and appetite scale, test drinks were consumed within a 5-min window, followed by a 120-min observation period (commenced upon the first ingested mouthful of the test drink). Blood samples were taken at 15, 30, 45, 60, 90 and 120-min following consumption of the test drinks. Appetite visual analogue scales were obtained at baseline and every 30 min throughout the postprandial period. Expired gas collection and blood pressure were taken at 60 and 120 min after test drink consumption.

### Blood sampling and analysis

A 10-mL blood sample was taken at each time point and allocated into tubes containing ethylenediaminetetraacetic acid (EDTA) (Sarstedt Ltd, Leicester, UK). Plasma samples were centrifuged immediately at 3465*g* at 4 °C for 10 min and stored at − 80 °C before performing analyses. Plasma glucose concentrations were determined using an automated analyzer (Daytona, Randox Laboratories) according to manufacturer’s instructions.

Plasma GLP-1_Total_, GIP_Total_ and PYY_Total_ were measured using commercially available enzyme-linked immunosorbent assays (ELISA; all from Merck Millipore Ltd. Watford, UK). The antibodies in the GLP-1 assay employed are specific to both GLP-1_7–36_ and GLP-1_9–36_ and therefore this assay captures GLP-1_Total_ concentrations. We assessed GLP-1_Total_, rather than GLP-1_7–36_, since this is the best indication of GLP-1 secretion in humans [3, 23]. Furthermore, the recovery is 90–110%, with a sensitivity of 1.5 pmol L^−1^, and there is no significant cross-reactivity with GLP-2, GIP, glucagon or oxyntomodulin [24]. The intra-assay and inter-assay coefficients of variation in our laboratory are < 9% and < 12%, respectively.

The GIP_Total_ assay has a sensitivity of 1.9 pmol L^−1^, intra-assay and inter-assay coefficients of variation of < 10% and < 10%, respectively. The PYY_Total_ assay has a sensitivity of 0.35 pmol L^−1^ intra-assay and inter-assay coefficients of variation of < 8% and < 12%, respectively.

### Expired breath analysis

Indirect calorimetry was performed using the Douglas bag method to assess energy expenditure and respiratory exchange ratio. A mouthpiece connected to a two-way, non-rebreathing valve (model 2730, Hans Rudolph, Kansas City, Missouri) was used to collect gas samples in Douglas bags, which were analyzed for concentrations of oxygen and carbon dioxide using paramagnetic and infrared transducers, respectively (Sevomex 5200S, Crowborough, East Sussex, UK). The ambient air was also analyzed at each time point to correct for changes in inspired gas concentrations [25]. Sensors were turned on 30 min prior to a two-point calibration (zero: 100% nitrogen; span: 16.93% oxygen and 5.04% carbon dioxide) using certified gases (BOC Industrial Gases, Linde AG, Munich, Germany).

Expired gas samples were corrected to standard temperature and pressure (dry) using a Fortin barometer (F.D. and company, Watford, UK). Volume and temperature of expired gas samples were determined using a dry gas meter (Harvard Apparatus, Edenbridge, Kent, UK) and thermistor (model 810-080, ETI, Worthing, UK), respectively, during gas evacuation.

### Blood pressure

Systolic and diastolic blood pressure were determined in triplicate using an automated blood pressure monitor, and the arm was standardized within participants (Panasonic EW3106 W, Osaka, Japan).

### Subjective appetite ratings

Subjective appetite was assessed using validated 100 mm visual analogue scales [26]. The questions asked were “how hungry do you feel” (with anchors: “I am not hungry at all” and “I have never been more hungry”), “how full do you feel” (with anchors: “Not at all full” and “Totally full”), “how satisfied do you feel” (with anchors: “I am completely empty” and “I cannot eat another bite”) and “how much do you think you can eat” (with anchors: “Nothing at all” and “A lot”). These were combined into an overall appetite rating, as previously described [27].

### Power calculations

Three sample size estimations were performed for the primary outcomes of each component study on the basis of GLP-1 responses as follows

#### Study 1: CALCITR versus MILK MINERALS versus MILK MINERALS + PROTEIN

Since it is hypothesised that calcium citrate should be absorbed in the intestine more proximally than calcium phosphate (and thus provide calcium to proximal versus distal components of the intestine), the sample size for study 1 was determined using plasma iAUC for GLP-1_7–36_ (in the absence of relevant data on GLP-1_Total_) in response to jejunal vs gastric feeding of mixed-macronutrients [28]. Jejunal vs gastric feeding produces a plasma GLP-1_7–36_ iAUC of 2.0 ± 1.4 vs 1.0 ± 1.3 mol L^−1^ × 720 min, respectively. Based on this effect size (*d*z = 0.83), 20 participants will provide > 80% power to statistically detect this effect with an α-level of 0.05 in a three-way crossover design.

This sample size was also deemed sufficient to detect changes between MILK MINERALS and MILK MINERALS + PROTEIN on the basis that protein delivered at a rate of 1.5 kcal min^−1^ to the duodenum results in a 60-min area under the curve for plasma GLP-1_Total_ of 1904 ± 138 pmol L^−1^ min, compared to 1490 ± 109 pmol L^−1^ min with saline control [29]. Based on this effect size (*d*z = 0.96), 20 participants should provide > 80% power to detect a similar effect size in a crossover design with three arms at an α-level of 0.05.

#### Study 2: PROTEIN versus MILK MINERALS + PROTEIN

The sample size estimation for the comparison of PROTEIN versus MILK MINERALS + PROTEIN was based on data from Mace et al. [8], where amino acid-induced GLP-1 secretion from an isolated rodent intestine is ~ 130 ± 127 pg mL (g dry weight)^−1 ^× min in low extracellular calcium concentrations [8]. When the extracellular calcium concentration is increased to that seen in the human ileum after a high-calcium meal [17], amino acid-induced GLP-1 secretion is increased to ~ 450 ± 71 pg mL (g dry weight)^−1 ^× min [8]. Using this effect size (d*z* = 3.11), four participants would provide greater than 80% power to detect this effect with an α-level of 0.05.

#### Study 3: CONTROL versus MILK MINERALS

The sample size estimation for the comparison of CONTROL versus MILK MINERALS was also based on data from Mace et al. [8], whereby, in the absence of amino acids and under conditions of low extracellular calcium concentrations, GLP-1 secretion is ~ 95 ± 28 pg mL (g dry weight)^−1^ min [17]. When the extracellular calcium concentration is increased to that seen in the human ileum after a high-calcium meal [17], GLP-1 secretion is increased to ~ 155 ± 42 pg mL (g dry weight)^−1^ min [8]. Using this effect size (d*z* = 1.68), 6 participants should provide greater than 80% power to detect such an effect with an α-level of 0.05.

### Statistical analysis

Statistical analyses were performed using GraphPad Prism v7 (GraphPad Software, San Diego, CA, USA). Values are mean ± SD in text, and means ± 95% confidence intervals in figures, unless stated otherwise. Due to technical issues in analysis, PYY data are *n* = 16 rather than *n* = 20 for study 1. Postprandial hormone and appetite responses were converted into incremental area under the curve (iAUC) and total area under the curve (AUC) using the trapezoidal rule. Data were checked for normal distribution by histograms of residuals and the Shapiro–Wilk normality test prior to analysis. There was no evidence of non-normal distribution and therefore parametric statistics were employed on all variables. Time-dependent variables were assessed by two-way (time × treatment) repeated-measures ANOVA. Differences between treatments in non-time-dependent variables (e.g., AUC and iAUC) were assessed by one-way ANOVA and Bonferonni-corrected *t* tests. Comparisons were considered to be significantly different when adjusted *p* values were ≤ 0.05. Since the studies were powered for gut hormone responses, and additional measures such as energy expenditure, appetite and blood pressure can be more variable, it was chosen to only present these additional data for study 1 (*n* = 20), due to a potential lack of power to make inferences regarding these variables in studies 2 and 3. However, these data are included in the online supplemental data (Supplementary Data File 1) for use in post-publication analyses such as meta-analyses. Some secondary outcomes were listed on the clinical trials registry, but were not analyzed due to lack of resource. These were plasma TAG, serum NEFA, and serum insulin concentrations.

## Results

### Study 1: MILK MINERALS + PROTEIN versus MILK MINERALS versus CALCITR

**Fig. 1:**
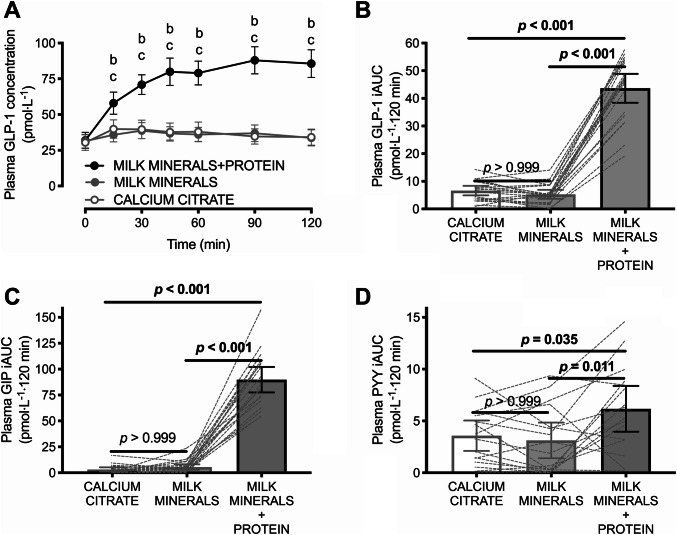
Plasma GLP-1 concentrations (**a**) and time-average incremental area under the curve (iAUC) values for plasma GLP-1 (**b**), GIP (**c**) and PYY (**d**) following ingestion of calcium citrate, milk minerals rich in calcium (MILK MINERALS) and MILK MINERALS plus whey protein hydrolysate (MILK MINERALS + PROTEIN) in healthy men and women. Data are means ± 95% CI, *n* = 20 for all data other than PYY, which are *n* = 16. *GLP-1* glucagon-like peptide-1, *GIP* glucose-dependent insulinotropic polypeptide, *PYY* peptide tyrosine tyrosine. ^b^Significant difference between MILK MINERALS + PROTEIN and MILK MINERALS; ^c^Significant difference between MILK MINERALS + PROTEIN and CITRATE (*p* ≤ 0.05)

Plasma GLP-1, GIP and PYY concentrations did not differ at baseline (all *p* > 0.999). Main effects of time and of treatment were both detected (both *p* < 0.001), in addition to a time × treatment interaction effect (*p* < 0.001) for the postprandial change in plasma GLP-1 (Fig. 1a). This resulted in a GLP-1 iAUC that was ~ ninefold higher with MILK MINERALS + PROTEIN compared to both CALCITR and MILK MINERALS (Fig. 1b; both *p* < 0.001). Peak GLP-1 concentrations reached 91 ± 20 pmol L^−1^ with MILK MINERALS + PROTEIN, compared to 43 ± 12 pmol L^−1^ and 46 ± 15 pmol L^−1^ with MILK MINERALS and CALCITR, respectively (both *p* < 0.001 versus MILK MINERALS + PROTEIN). Peak GIP concentrations reached 177 ± 45 pmol L^−1^ with MILK MINERALS + PROTEIN, compared to 54 ± 25 pmol L^−1^ and 52 ± 25 pmol L^−1^ with MILK MINERALS and CALCITR, respectively (both *p* < 0.001 versus MILK MINERALS + PROTEIN). Peak PYY concentrations reached 93 ± 55 pmol L^−1^ with MILK MINERALS + PROTEIN, compared to 81 ± 46 pmol L^−1^ and 87 ± 50 pmol L^−1^ with MILK MINERALS (*p* < 0.02) and CALCITR (*p* = 0.64), respectively. Furthermore, the iAUC for GIP (~ 21-fold, Fig. 1c; both *p* < 0.001) and PYY (~ twofold, Fig. 1d; both *p* < 0.04) were also higher with MILK MINERALS + PROTEIN compared to both CALCITR and MILK MINERALS. No difference in the plasma GLP-1, GIP and PYY iAUCs were detected between MILK MINERALS and CALCITR (Fig. 1b, *p* > 0.999; Fig. 1c, *p* > 0.99 and Fig. 1d, *p* > 0.999, respectively).

**Fig. 2:**
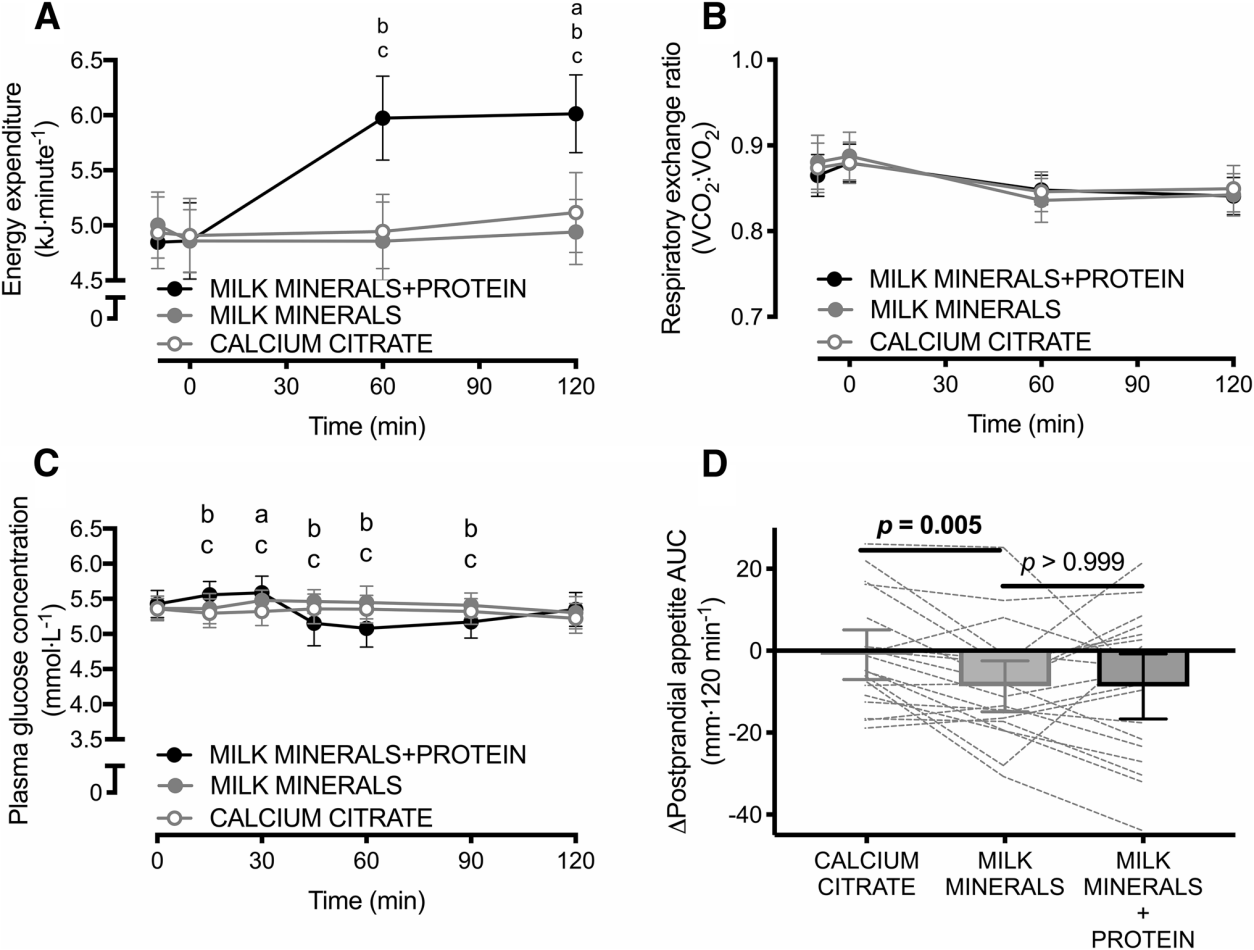
Energy expenditure (**a**), respiratory exchange ratio (**b**), plasma glucose concentrations (**c**) and time-average postprandial area under the curve (AUC) values for appetite (**d**) following ingestion of calcium citrate, milk minerals rich in calcium (MILK MINERALS) and MILK MINERALS plus whey protein hydrolysate (MILK MINERALS + PROTEIN) in healthy men and women. Data are means ± 95% CI, *n* = 20. ^a^Significant difference between CITRATE and MILK MINERALS; ^b^Significant difference between MILK MINERALS + PROTEIN and MILK MINERALS; ^c^Significant difference between MILK MINERALS + PROTEIN and CITRATE (*p* ≤ 0.05)

Energy expenditure did not differ at baseline (*p* = 0.14). There was a main effect of time, a main effect of treatment, and a time × treatment interaction effect for energy expenditure (all *p* < 0.001), whereby MILK MINERALS + PROTEIN increased postprandial energy expenditure by 1.1 ± 0.4 kJ min^−1^ and 1.0 ± 0.5 kJ min^−1^ compared to both MILK MINERALS and CALCITR (Fig. 2a, both *p* < 0.001; representing an increase of ~ 21%). A main effect of time was also detected for the respiratory exchange ratio (Fig. 2b; *p* < 0.001), but no main effect of treatment, nor any time × treatment interaction effect was observed for the respiratory exchange ratio (both *p* > 0.5).

Plasma glucose concentrations did not differ at baseline between any treatment (all *p* > 0.8). Following ingestion of the test drinks, plasma glucose concentrations demonstrated a main effect of time (*p* < 0.001), and a time × treatment interaction effect (*p* < 0.001), whereby plasma glucose initially increased, and then decreased with MILK MINERALS + PROTEIN compared to CALCITR and MILK MINERALS (Fig. 2c). However, absolute differences in glucose concentrations were not of a magnitude that is biologically important (maximal difference: − 0.37 ± 0.51 mmol L^−1^) and plasma glucose concentrations remained within a relatively tight range across all conditions.

Baseline appetite ratings were higher with MILK MINERALS versus CALCITR (71 ± 14 versus 66 ± 14 au; *p* = 0.044), but did not differ between MILK MINERALS + PROTEIN (70 ± 16 au) versus either MILK MINERALS or CALCITR (both *p* > 0.258). A main effect of time (*p* < 0.0001) and a trend for time × treatment interaction effect (*p* = 0.054) were detected for postprandial appetite ratings. Accordingly, the postprandial suppression of appetite expressed as an AUC was greater with MILK MINERALS versus CALCITR (Fig. 2d; *p* = 0.005). No difference in the postprandial suppression of appetite expressed as an AUC was detected between MILK MINERALS and MILK MINERALS + PROTEIN (Fig. 2d; *p* > 0.999).

**Fig. 3:**
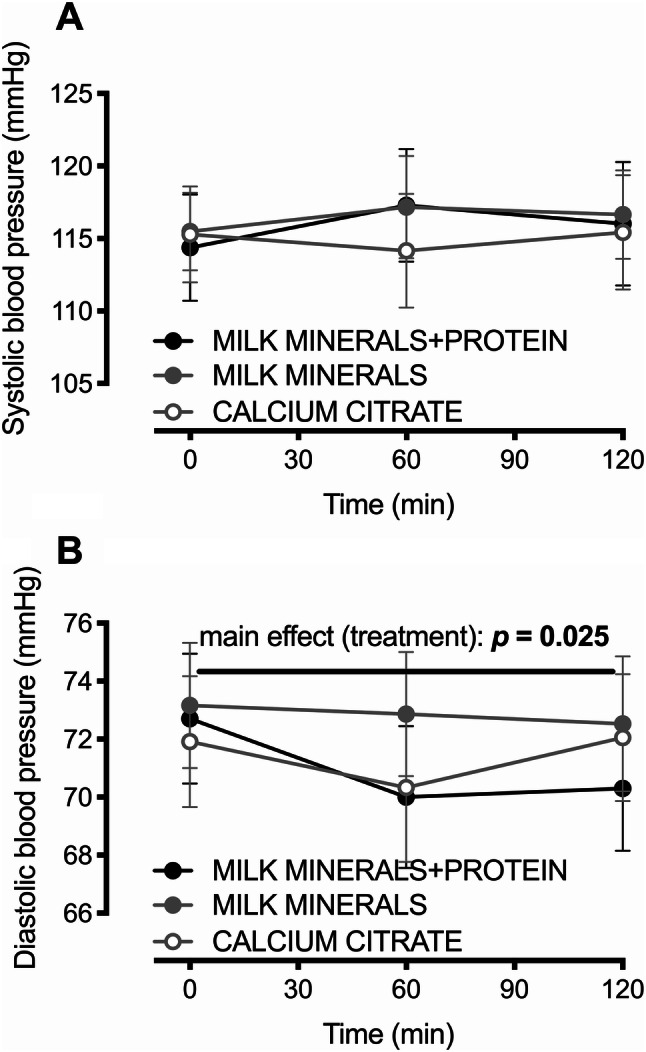
Systolic (**a**) and diastolic (**b**) blood pressure following ingestion of calcium citrate, milk minerals rich in calcium (MILK MINERALS) and MILK MINERALS plus whey protein hydrolysate (MILK MINERALS + PROTEIN) in healthy men and women. Data are means ± 95% CI, *n* = 20

Neither systolic blood pressure, nor diastolic blood pressure differed at baseline between treatments (all *p* > 0.6). No main effects of time or treatment, nor any time × treatment interaction effects were observed for systolic blood pressure (all *p* > 0.3; Fig. 3a). Diastolic blood pressure, however, displayed both a main effect of time (*p* = 0.033) and a main effect of treatment (*p* = 0.025), whereby the MILK MINERALS + PROTEIN produced a diastolic blood pressure than was 1.9 mmHg (95% CI 0.2–3.6 mmHg) lower than MILK MINERALS (Fig. 3b; *p* = 0.092).

### Study 2: MILK MINERALS + PROTEIN versus PROTEIN

**Fig. 4:**
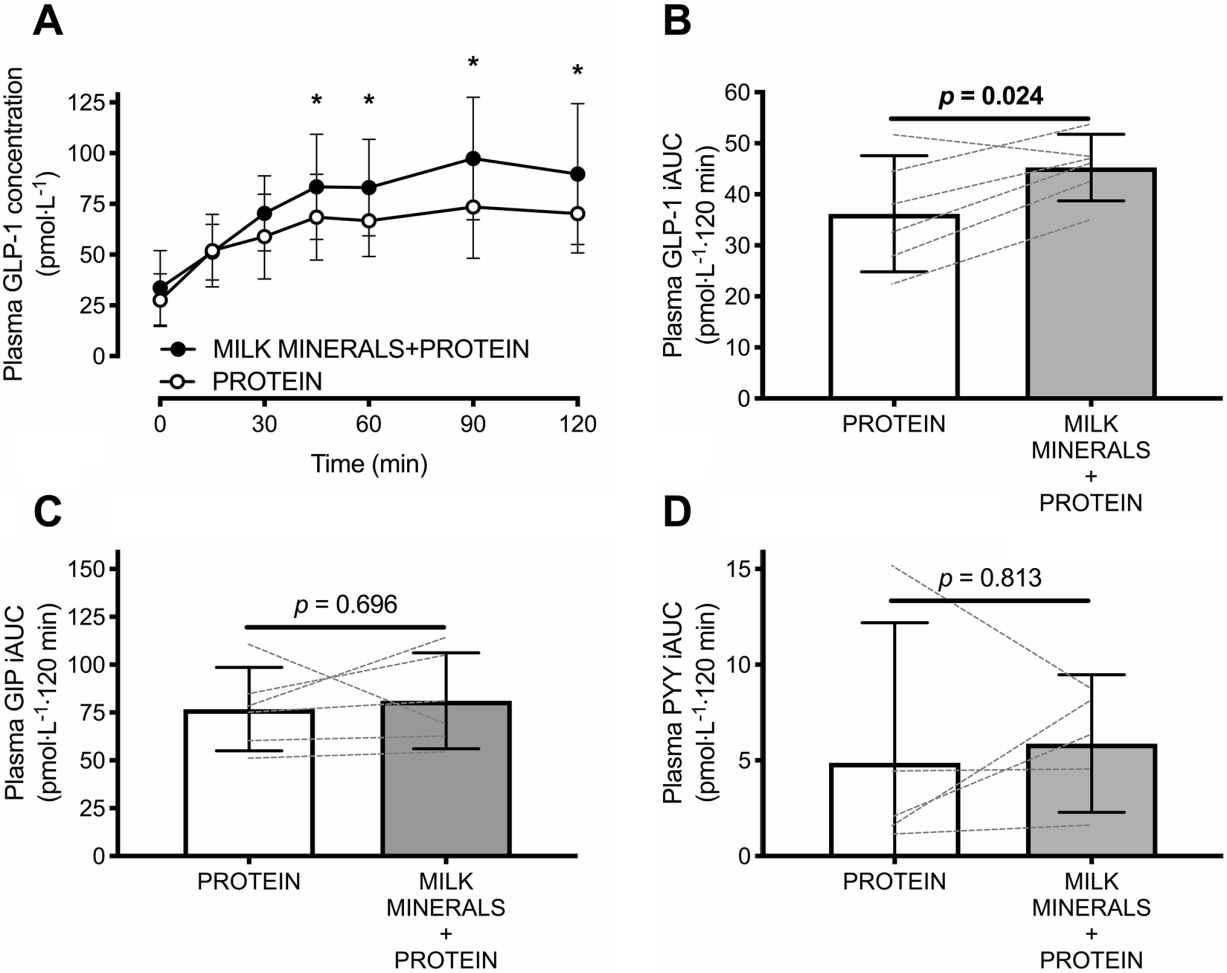
Plasma GLP-1 concentrations (**a**) and time-average incremental area under the curve (iAUC) values for plasma GLP-1 (**b**), GIP (**c**) and PYY (**d**) following ingestion of whey protein hydrolysate in the presence (MILK MINERALS + PROTEIN) and absence (PROTEIN) of milk minerals rich in calcium in healthy men and women. Data are means ± 95% CI, *n* = 6. *GLP-1* glucagon-like peptide-1, *GIP* glucose-dependent insulinotropic polypeptide, *PYY* peptide tyrosine tyrosine. *Significant difference between treatments (*p* ≤ 0.05)

Plasma GLP-1 and GIP concentrations did not differ at baseline (both *p* > 0.99, but plasma PYY concentrations were higher with PROTEIN versus MILK MINERALS + PROTEIN (23 ± 6 versus 15 ± 3 pmol L^−1^; *p* = 0.004). A time × treatment interaction effect was detected (*p* = 0.037) for postprandial plasma GLP-1 concentrations (Fig. 4a). This resulted in a plasma GLP-1 iAUC that was ~ 25% higher with MILK MINERALS + PROTEIN compared to PROTEIN (Fig. 4b; *p* = 0.024; mean difference: 9.1 ± 6.9 pmol L^−1^ 120 min). A main effect of time was detected for the postprandial change in plasma GIP and PYY concentrations (both, *p* < 0.001), but no main effect of treatment, nor time × treatment interaction effect was observed (all *p* > 0.05). These resulted in a plasma GIP iAUC (Fig. 4c; *p* = 0.696) and PYY iAUC (Fig. 4d; *p* = 0.438) that did not differ between MILK MINERALS + PROTEIN and PROTEIN.

### Study 3: MILK MINERALS versus CONTROL

**Fig. 5:**
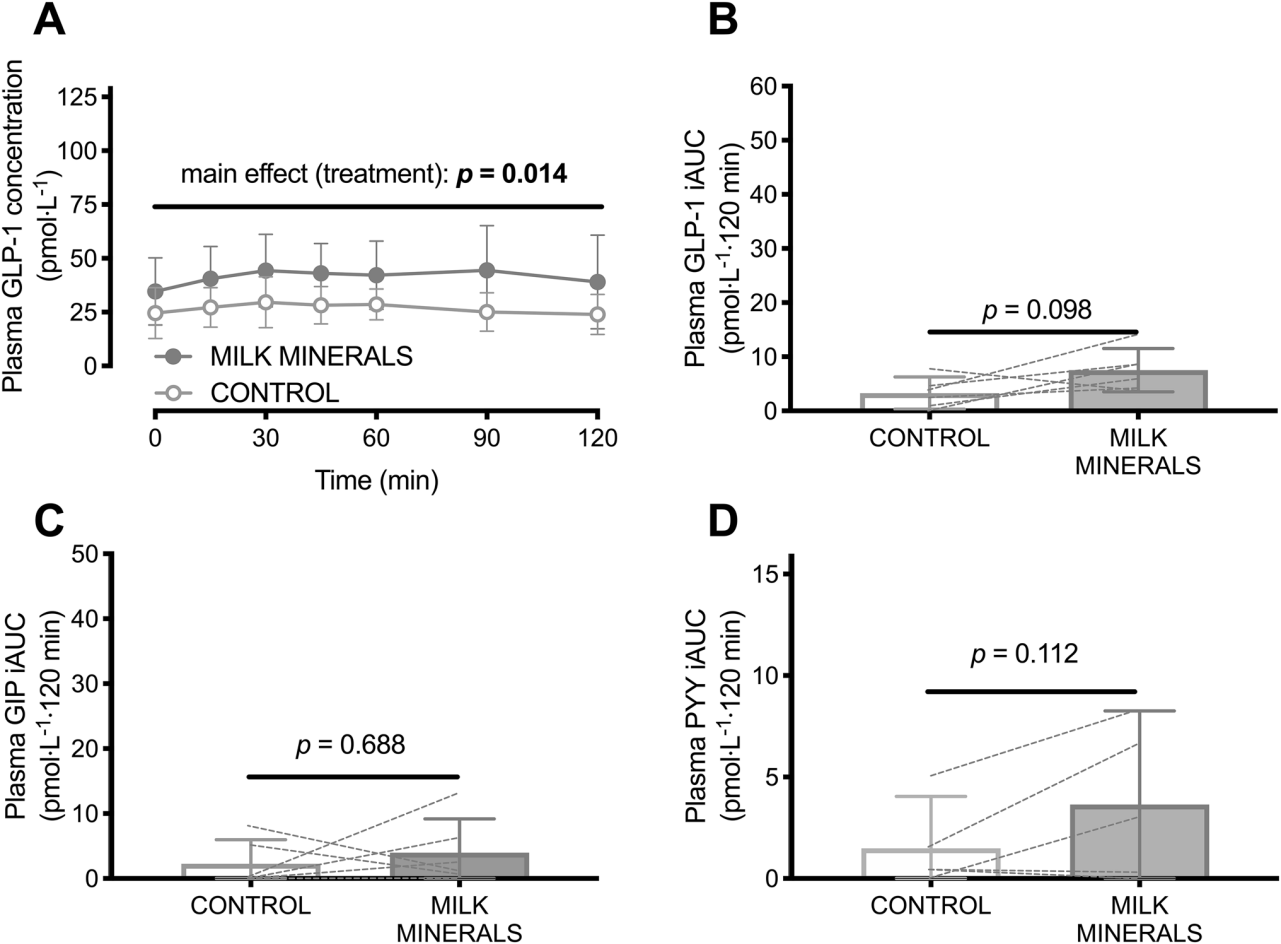
Plasma GLP-1 concentrations (**a**) and time-average incremental area under the curve (iAUC) values for plasma GLP-1 (**b**), GIP (**c**) and PYY (**d**) following ingestion of milk minerals rich in calcium (MILK MINERALS) or the vehicle of ingestion (CONTROL; 500 mL water plus 80 mg sucralose) in healthy men and women. Data are means ± 95% CI, *n* = 6. *GLP-1* glucagon-like peptide-1, *GIP* glucose-dependent insulinotropic polypeptide, *PYY* peptide tyrosine tyrosine

Baseline GLP-1 concentrations (but not GIP, nor PYY concentrations) were higher in the MILK MINERALS trial *versus* CONTROL (36 ± 15 versus 25 ± 11 pmol L^−1^; *p* = 0.02). There was a main effect of treatment (*p* = 0.01), but no main effects of time (*p* = 0.07) nor a time × treatment interaction effect (*p* = 0.58) for postprandial plasma GLP-1 concentrations (Fig. 5a). The increase in the plasma iAUC with MILK MINERALS versus CONTROL was therefore trivial (Fig. 5b; *p* = 0.098; mean difference: 4.2 ± 5.1 pmol L^−1^ 120 min). A main effect of time was detected for the postprandial change in plasma GIP concentrations (*p* = 0.04), but no main effect of treatment, or time × treatment interaction effect was observed (both *p* > 0.7). No main effects of time or treatment, nor time × treatment interaction effect was observed in plasma PYY (all *p* > 0.1). Accordingly, no difference was detected in the plasma iAUC for GIP (Fig. 5c; *p* = 0.688) and PYY (Fig. 5d; *p* = 0.112) between MILK MINERALS and CONTROL.

## Discussion

The present work demonstrates that co-ingestion of whey protein hydrolysate with milk minerals rich in calcium potently stimulates plasma GLP-1 availability. Furthermore, milk minerals rich in calcium appear to suppress appetite ratings to a greater extent than an equivalent quantity of calcium from calcium citrate when ingested in isolation. Finally, ingestion of whey protein hydrolysate plus milk minerals rich in calcium, but not calcium alone, acutely reduces diastolic blood pressure.

There are mounting rodent data suggesting that calcium plays an important role in amino acid-induced gut hormone secretion. Ex vivo intestinal perfusion demonstrates that the calcium concentration of the environment is essential for robust amino acid-induced GLP-1 secretion [8], which is thought to act via the synergistic stimulation of the calcium-sensing receptor by both calcium and amino acids [10, 30]. It has also been demonstrated more recently that the calcium-sensing receptor mediates the effects of amino acids on gut hormone secretion, appetite and food intake in rodents, in vivo [11]. The present study is the first to demonstrate that ingestion of milk minerals rich in calcium further increases whey protein-induced postprandial GLP-1 secretion in humans. Increasing postprandial GLP-1 concentrations is a key target for improving metabolic health and/or decreasing obesity risk by stimulating insulin secretion and angiogenesis, whilst suppressing appetite and energy intake [1–3].

The postprandial GLP-1 concentrations in the current study are some of the highest ever reported in physiological conditions. Importantly, fasting concentrations were in line with those reported by others in similar healthy populations, typically between 20 and 40 pmol L^−1^ [29, 31, 32], yet the combined ingestion of ~ 1000 mg milk minerals rich in calcium with 50 g whey protein hydrolysate produced remarkably high plasma GLP-1 concentrations. The mean peak postprandial concentrations (91 ± 20 pmol L^−1^) are more than double that reported with whey protein ingestion in a similar healthy population, even with 70 g whey protein isolate ingested orally [31], or 50 g administered via intraduodenal infusion [29]. Furthermore, peak GLP-1 concentrations with Roux-en-Y gastric bypass surgery have been shown to increase from ~ 20 pmol L^−1^ pre-surgery, to ~ 100 pmol L^−1^ post-surgery [33]. The dose of protein used in the present study is still relatively high from a practical perspective, and therefore the effects of protein and calcium co-ingestion should be explored with lower doses in the future. Nevertheless, the postprandial plasma concentrations of GLP-1 that we report are therefore some of the highest in the literature with physiological ingestion of nutrients.

The reasons for the remarkably high GLP-1 concentrations observed here could be due to: (1) the GLP-1 assay method, (2) the use of arterialized blood sampling, and/or (3) the co-ingestion of calcium with whey protein hydrolysate. First, the assay employed in the present study has been shown to have good precision and specificity for GLP-1_Total_ [24]. Furthermore, the basal concentrations we report are in line with previous studies in similar cohorts [29, 31], and ELISAs can tend to under-estimate GLP-1 concentrations compared to radioimmunoassays [24]. Therefore, the high concentrations we report are not likely to be an artefact of the assay employed.

Regarding the second possibility, GLP-1 concentrations are higher in arterial, compared to venous blood [34, 35], presumably due to tissue uptake or binding with GLP-1 receptors. Therefore, as with glycaemia [22], it may be recommended to sample from arterial or arterialized blood when systemic postprandial gut hormone concentrations require accurate quantification. However, the difference in peak postprandial GLP-1_Total_ concentrations between arterialized and venous blood is ~ 10 pmol L^−1^ [35]. Consequently, the blood sampling method employed can only explain a small fraction of the remarkably high GLP-1 concentrations that we report. It is, therefore, highly likely that the primary reason for the high GLP-1 concentrations in the present study is the potency of the milk minerals rich in calcium plus whey protein hydrolysate test-drink.

Theoretically, the exceptionally high concentrations of GLP-1 reported in the present study could be due to the ingestion of whey protein hydrolysate increasing intestinal K- and L-cell exposure to amino acids to a greater extent than other (non-hydrolysed) protein sources that have previously been investigated [30]. However, the ingestion of 50 g albumin has been shown to increase ileal concentrations of free amino acids to ~ 20 mmol L^−1^ [36], which is already ~ twofold higher than the concentration thought to maximize GLP-1 secretion in the presence or absence of calcium [8]. Therefore, any potential further increase in free amino acid availability with whey protein hydrolysate is unlikely to further stimulate GLP-1 secretion and thus, the co-ingestion of calcium with protein is the most likely explanation for the exceptionally high postprandial GLP-1 concentrations we report. To confirm this, we performed a second study, which demonstrated that the addition of milk minerals rich in calcium to whey protein hydrolysate further increases postprandial GLP-1 concentrations by ~ 25%. Furthermore, the calcium-induced increase in GLP-1 concentrations was more than two-fold in magnitude when ingested with *versus* without protein (iAUC ~ 9 versus ~ 4 pmol L^−1^ 120 min). Whilst this requires confirmation as this was not a direct (within-subject) comparison, this suggests that the ability of milk minerals rich in calcium to influence GLP-1 secretion may be partly dependent on co-ingestion of protein (or other macronutrients). Interestingly, such a synergistic effect was not observed for GIP, nor PYY concentrations, highlighting a potentially important role of calcium in GLP-1 secretion. It remains to be seen whether milk sources of calcium (or minor quantities of other minerals present in the milk mineral mixture enriched in calcium), are of particular importance in GLP-1 secretion.

The ingestion of milk minerals rich in calcium suppressed postprandial subjective appetite ratings to a greater extent than calcium citrate, although this may have been partly driven by baseline differences between these two conditions. Interestingly, there was no further suppression of appetite by the addition of 50 g whey protein hydrolysate to milk minerals rich in calcium ingestion. We have previously reported that milk minerals rich in calcium suppresses appetite ratings and energy intake independent of milk protein [7]. The present data confirm this response and extend it to whey protein hydrolysate, rather than milk minerals rich in calcium alone. The mechanisms for the suppression in subjective appetite ratings are unclear and cannot be explained by changes in GLP-1 or PYY concentrations. Whilst GLP-1 and PYY contribute to appetite regulation, it is possible that other mechanisms such as alterations in gastric emptying, or other gut hormones such as cholecystokinin or OXM could be influenced by calcium ingestion in a way that would suppress appetite and supersede the changes in GLP-1 observed in the present study. These potential mechanisms require further exploration. There is also a need to understand whether these changes in gut hormone availability translate into changes in insulinaemia and/or ad libitum energy intake (sufficient to offset the higher energy load of protein co-ingestion).

Whey protein hydrolysate also produced a robust increase in energy expenditure. Protein stimulates postprandial thermogenesis to a greater extent than other macronutrients, and whey protein stimulates postprandial thermogenesis more than other protein sources [37]. The increase in energy expenditure in the current study is in close agreement (~ 1 kJ min^−1^, equivalent to ~ 16 kcal per hour) with that reported by others [37]. Therefore, the combination of milk minerals rich in calcium with whey protein hydrolysate produces a scenario of enhanced GLP-1 secretion, suppressed appetite ratings, and increased resting energy expenditure (albeit the increase in resting energy expenditure is modest in the context of energy balance). Since some gut hormones are also implicated in physical activity energy expenditure [38], it remains to be seen whether this nutritional combination also stimulates physical activity energy expenditure (and/or affects energy intake), thereby further contributing to weight control and metabolic health.

Both dietary calcium and whey protein have been implicated in blood pressure reduction, albeit with an equivocal evidence base [39, 40]. Here we demonstrate that neither calcium citrate nor milk sources of calcium acutely alter blood pressure, whereas whey protein hydrolysate plus milk minerals rich in calcium acutely reduces diastolic blood pressure by ~ 2 mmHg. It should be noted that the present study design did not contain an isocaloric comparator to whey protein hydrolysate ingestion, and it is unknown whether the responses to protein ingestion were macronutrient-specific or due to the higher energy load in that trial. Furthermore, heart rate was not measured in the present study, and therefore it is not possible to assess whether a compensatory increase in heart rate was observed in response to the lowering of blood pressure. Nevertheless, other work has demonstrated that whey protein ingestion can acutely lower systolic blood pressure compared to isocaloric maltodextrin ingestion, without any evidence of compensatory increases in heart rate [39]. Longer-term studies are required to assess whether this acute 2 mmHg reduction in blood pressure translates into chronic changes in blood pressure of a meaningful magnitude.

In conclusion, this collective series of studies is the first to show that whey protein hydrolysate potently stimulates GLP-1 secretion, and that this response can be further enhanced (albeit modestly) by the co-ingestion of dietary calcium. It remains to be established if this is a protein-specific (or energy-specific) response. Nevertheless, this suggests that the addition of calcium to protein ingestion can potentiate postprandial GLP-1 concentrations without further increasing the energy load. In addition, these data demonstrate that milk minerals enriched in calcium suppress appetite to a greater extent than calcium citrate and this suppression does not require the addition of protein. Finally, dietary calcium does not appear to acutely affect blood pressure in healthy men and women.

## Electronic supplementary material

Below is the link to the electronic supplementary material.

Supplementary material 1 (DOC 218 kb)

Supplementary material 2 (DOC 57 kb)

Supplementary material 3 (CSV 55 kb)

## Abbreviations

GLP-1: Glucagon-like peptide-1
GIP: Glucose-dependent insulinotropic polypeptide
PYY: Peptide tyrosine–tyrosine
iAUC: Incremental area under the curve
