# Supplementary material for: Co-ingestion of whey protein hydrolysate with milk minerals rich in calcium potently stimulates glucagon-like peptide-1 secretion: an RCT in healthy adults
Source: Eur J Nutr. 2019 Sep 17;59(6):2449–62. doi: 10.1007/s00394-019-02092-4 (PMC7413905; doi:10.1007/s00394-019-02092-4)
Supplement: Supplementary file 2 — Supplementary material 2 (DOC 57 kb) [file 394_2019_2092_MOESM2_ESM.doc]

**
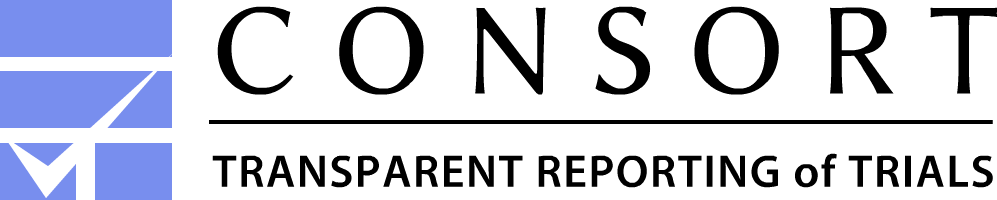
**

**CONSORT 2010 Flow Diagram**

**Allocation**

**Analysis**

**Follow-Up**

**Enrollment**

**Analysis**

**Follow-Up**

**Analysis**

**Follow-Up**

**Analysis**

**Follow-Up**

Assessed for eligibility (n= 21)

Excluded (n=1)

♦  Not meeting inclusion criteria (n=0)

♦  Declined to participate (n=0)

♦  Other reasons (n=1 drop-out prior to randomization due to ongoing headaches)

Analysed (n=12)
♦ Excluded from analysis (give reasons) (n=0)

Lost to follow-up (give reasons) (n=0)

Discontinued intervention (give reasons) (n=0)

Allocated to treatments CALCITR, MILK MINERALS, and MILK MINERALS+PROTEIN (n=12)

♦ Received allocated intervention (n=12)

♦ Did not receive allocated intervention (give reasons) (n=0)

Randomized (n= 20)

Allocated to treatments CALCITR, MILK MINERALS, MILK MINERALS+PROTEIN, PROTEIN and CONTROL(n=4)

♦ Received allocated intervention (n=4)

♦ Did not receive allocated intervention (give reasons) (n=0)

Allocated to treatments CALCITR, MILK MINERALS, and MILK MINERALS+PROTEIN, and PROTEIN (n=2)

♦ Received allocated intervention (n=2)

♦ Did not receive allocated intervention (give reasons) (n=0)

Allocated to treatments CALCITR, MILK MINERALS, and MILK MINERALS+PROTEIN, and CONTROL (n=2)

♦ Received allocated intervention (n=2)

♦ Did not receive allocated intervention (give reasons) (n=0)

Analysed (n=4)
♦ Excluded from analysis (give reasons) (n=0)

Lost to follow-up (give reasons) (n=0)

Discontinued intervention (give reasons) (n=0)

Analysed (n=2)
♦ Excluded from analysis (give reasons) (n=0)

Lost to follow-up (give reasons) (n=0)

Discontinued intervention (give reasons) (n=0)

Analysed (n=2)
♦ Excluded from analysis (give reasons) (n=0)

Lost to follow-up (give reasons) (n=0)

Discontinued intervention (give reasons) (n=0)
